# Isolated Pancreatic Metastasis in a Patient With Orbital Rhabdomyosarcoma: A Report of a Rare Case

**DOI:** 10.7759/cureus.70770

**Published:** 2024-10-03

**Authors:** Rana Bilal Idrees, Mariam Malik, Ahmed Mustanser, Taimoor Sarwar, Farzana kousar, Muhammad Hamid Chaudhary

**Affiliations:** 1 Radiology, Institute of Nuclear Medicine & Oncology Lahore Cancer Hospital, Lahore, PAK; 2 Radiology, Atomic Energy Cancer Hospital, Nuclear Medicine, Oncology and Radiotherapy Institute, Islamabad, PAK; 3 Nuclear Medicine, Institute of Nuclear Medicine & Oncology Lahore Cancer Hospital, Lahore, PAK; 4 Cardiac Surgery, Chaudhry Pervaiz Elahi Institute of Cardiology, Multan, PAK

**Keywords:** alveolar rhabdomyosarcoma, eye rhabdomyosarcoma, orbital rhabdomyosarcoma, pancreatic metastasis, soft-tissue sarcoma

## Abstract

Rhabdomyosarcoma (RMS) is the most common soft tissue sarcoma in children and adolescents and its occurrence in adults is extremely rare. There are three major subtypes of RMS of which alveolar RMS (ARMS) has the worst prognosis and tends to metastasize to unusual locations such as the pancreas. We present a case of a 19-year-old male with a rapidly enlarging right orbital mass, the imaging of which revealed it to be an infiltrative lesion with extension into the nasal cavity, ethmoid sinuses, and intracranial involvement. Histopathology confirmed the diagnosis of ARMS. A staging CT scan showed an enlarged and hypoenhancing pancreas; the histopathology test confirmed ARMS metastasis.

It is important to consider pancreatic involvement in patients with ARMS, as it may be misinterpreted with other pathologies such as pancreatitis and pancreatic lymphoma. Metastasis to the pancreas can significantly alter the clinical approach and staging, underscoring the need for accurate diagnosis and staging in these patients.

## Introduction

Sarcomas account for less than 1% of all adult solid tumors, and only 3% of these are rhabdomyosarcomas (RMSs) [1]. While RMS is the most common soft tissue sarcoma in children and adolescents, its occurrence in adults is exceedingly rare [2]. RMS is classified into three major subtypes: embryonal (60%), alveolar (20%), and undifferentiated or miscellaneous types. The embryonal and alveolar subtypes are frequently seen in children, but can also be present in adults, with the latter subtypes being more commonly encountered in adults. The most frequent locations for RMS are the head and neck, accounting for 30-40% of cases, followed by the genitourinary tract and extremities [3]. In adults, 19-24% of RMS cases involve the head and neck regions [4].

Orbital RMS arises from striated muscle cells or their mesenchymal precursors and is a rare tumor primarily seen in children, with an average age of presentation between seven and eight years. In adults, orbital RMS is extremely rare and tends to be aggressive. By the time of diagnosis, more than half of adult patients present with regional or distant metastases, commonly affecting the lungs, bones, bone marrow, and lymph nodes. The treatment typically involves a multimodal approach, including radiotherapy, but distant metastasis drastically worsens the prognosis, with a five-year survival rate of less than 20% [2].

Pancreatic metastases occur in 3-12% of patients with widespread metastatic disease at autopsy [5]. However, isolated pancreatic metastasis from RMS is exceedingly rare, with only a few cases reported in the literature [6]. Recognizing this unusual presentation is critical, as it necessitates a more aggressive therapeutic approach. In this report, we present a rare case of alveolar RMS of the right orbit with isolated pancreatic metastasis in an adult patient.

## Case presentation

A 19-year-old male patient presented with a three-month history of a rapidly enlarging right orbital mass. On clinical examination, there was right-sided proptosis associated with chemosis, impaired vision, ophthalmoplegia, and right nasal stuffiness.

**Figure 1:**
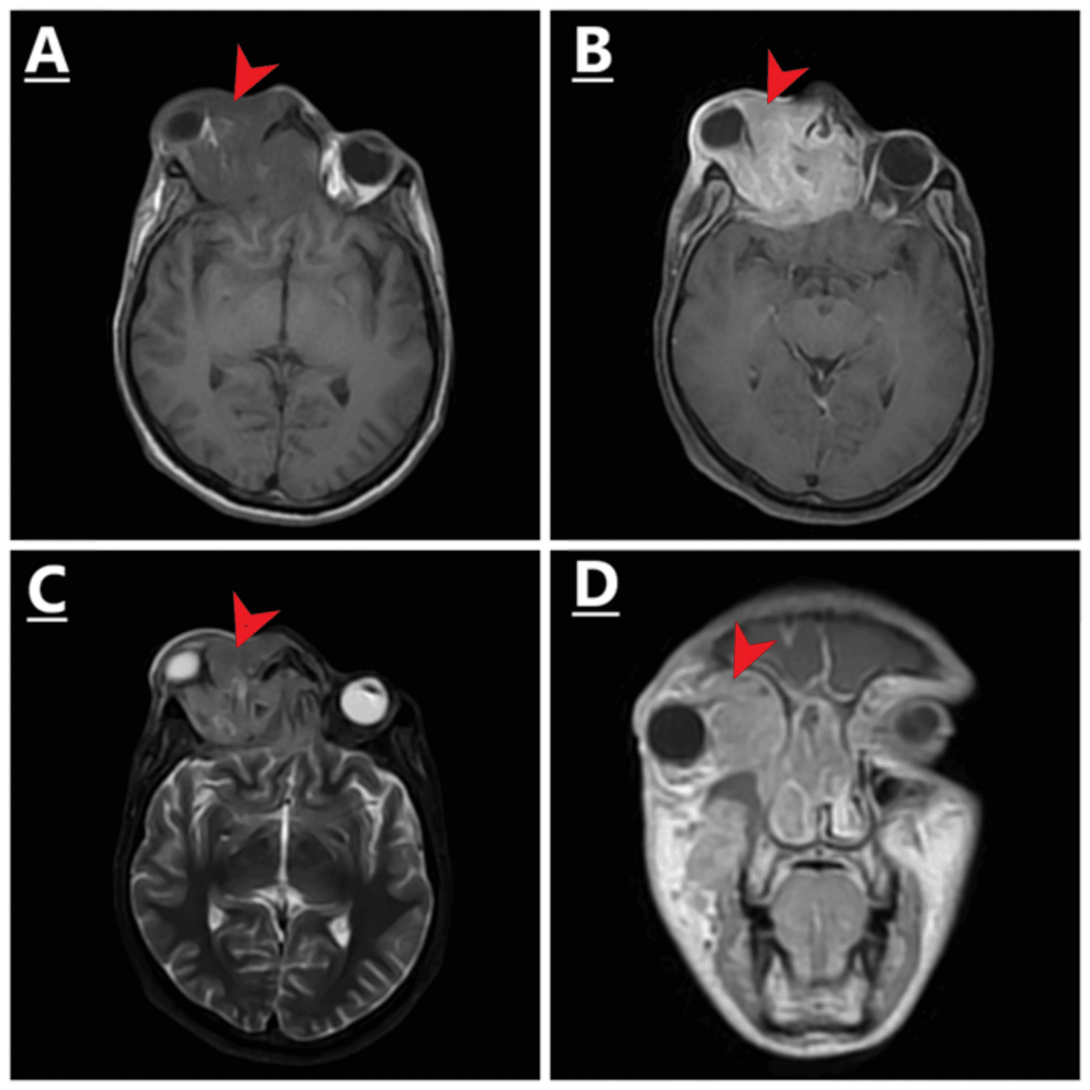
The mass is annotated by red arrows. A) Non-contrast axial T1W demonstrating a hypointense lesion arising from the right orbit with extension into the ipsilateral half of the nasal cavity and right ethmoid sinuses. B) Contrast-enhanced axial T1W showing post-contrast enhancement of the mass. C) Axial short TI inversion recovery (STIR). D) Coronal T1 post-contrast images demonstrating the infiltrative nature of the disease process.

Contrast-enhanced MRI of the head and neck showed an irregular ill-defined infiltrative mass involving the right orbit extending into the right nasal cavity and right ethmoid sinuses. There was an intracranial extra-axial extension of the disease with a mass effect on the ipsilateral basifrontal lobe (Figure 1). Histopathology of the orbital mass confirmed the diagnosis of alveolar RMS (ARMS).

**Figure 2:**
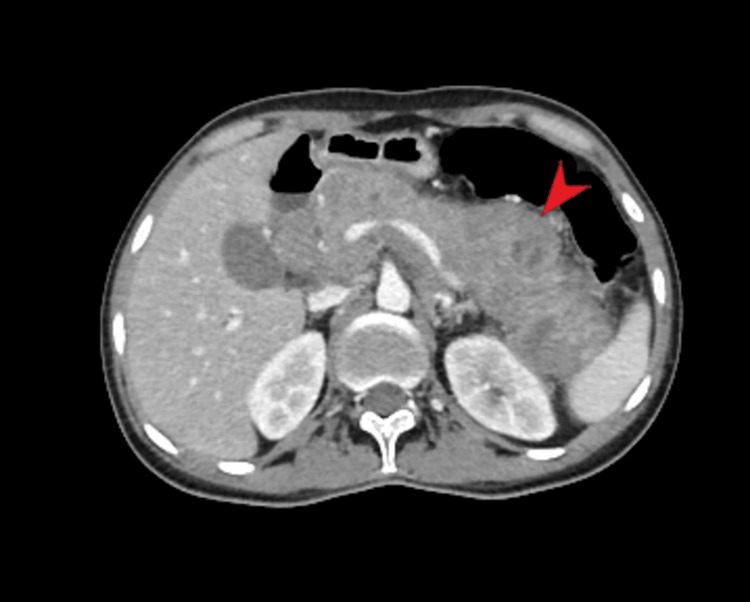
Selected axial post-contrast contrast-enhanced CT (CECT) section showing diffusely swollen heterogeneous relatively hypoenhancing pancreas annotated by the red arrow.

A staging contrast-enhanced CT (CECT) neck, chest, abdomen, and pelvis showed an enlarged swollen, relatively hypoenhancing pancreas, while the rest of the study was unremarkable for metastasis (Figure 2).

Based on the isolated pancreatic findings, differential diagnoses of pancreatitis, pancreatic lymphoma, and metastasis from the primary orbital ARMS were given. Laboratory correlation revealed serum amylase and lipase to be marginally elevated while IgG4 and antinuclear antibody (ANA) levels were found to be normal (Table 1).

**Table 1:** Parameters, patient values, and the reference range of each parameter are presented in a tabulated form.

Parameter	Patient’s value	Reference range
Serum amylase	135 units per liter (U/L)	30 to 110 units per liter (U/L)
Serum lipase	169 units per liter (U/L)	0 to 160 units per liter (U/L)
Serum IgG4	15 mg/dL	Less than 10 mg/dL to 140 mg/dL
Antinuclear antibodies	Less than 1.0 (non-reactive)	Less than or equal to I.0 U is negative. 1.1-2.9 U is weakly positive. 3.0-5.9 U is positive. Greater than or equal to 6.0 U is strongly positive

**Figure 3:**
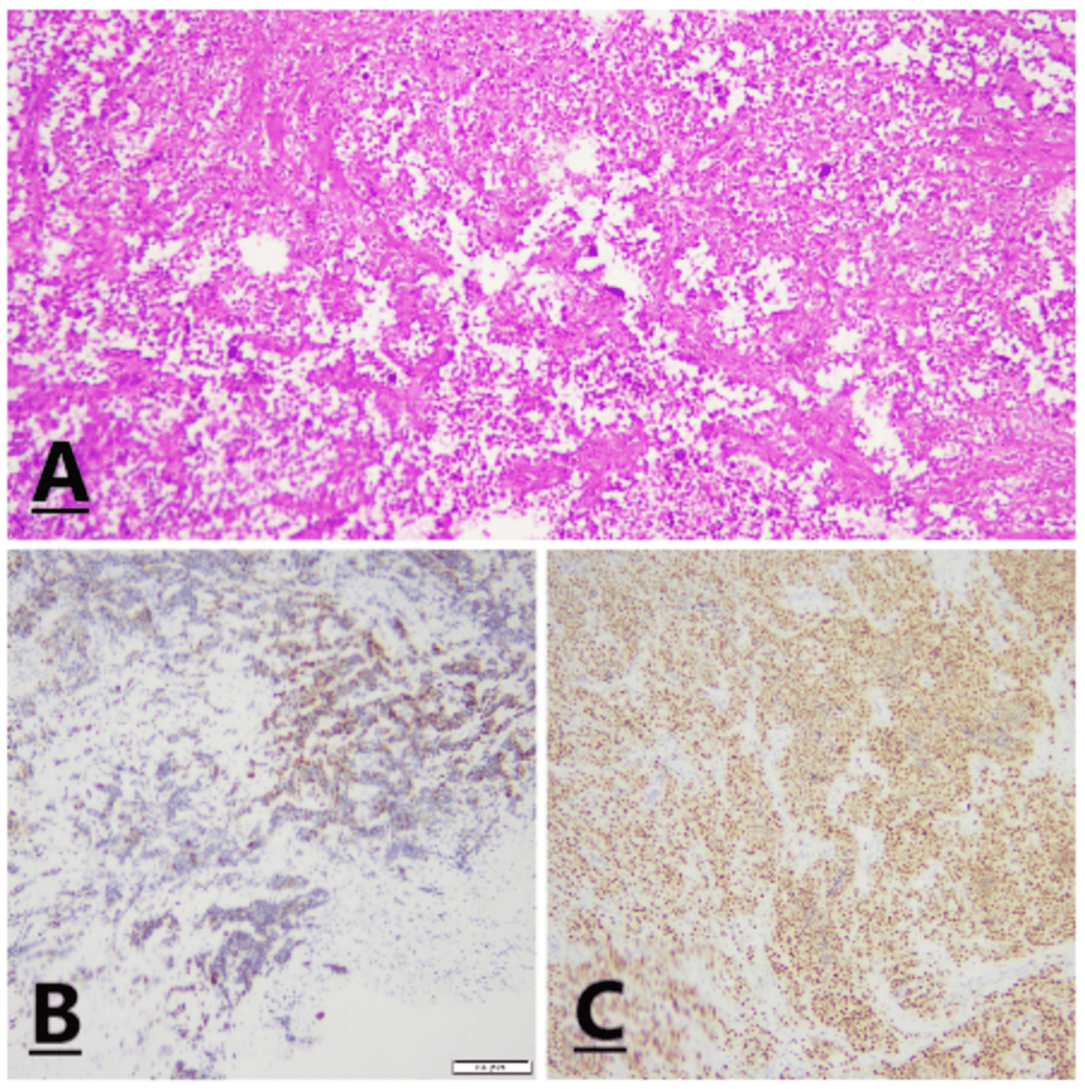
A) Histological examination shows an infiltrative cellular tumor composed of large clusters and nests of primitive round cells, separated by variably thick fibrovascular septa. Irregular pseudoalveolar spaces and cystic change are present within the nests. B and C) Immunohistochemistry (IHC) shows tumor cells are positive for desmin (B) with strong diffuse myogenin expression (C).

Histopathological correlation was advised for definite characterization. A core biopsy performed under ultrasound guidance confirmed metastasis of ARMS (Figure 3).

The presence of metastasis to the pancreas upstaged the disease to stage IV excluding surgery from the management plan. Hence the patient was referred to the oncology department for treatment with palliative intent. The patient underwent three sessions of chemotherapy; however, unfortunately, the disease progressed leading to the death of the patient. 

## Discussion

Understanding both typical and atypical presentations of orbital RMS is essential for early diagnosis and treatment. Symptoms in patients with orbital RMS can range from an eyelid nodule to proptosis, which may be misinterpreted as inflammatory or vascular lesions such as hemangiomas, underscoring the necessity of a biopsy for definitive diagnosis [3]. Differential diagnoses include orbital cellulitis, dermoid cysts, lymphoma, orbital pseudotumor, orbital metastasis, and lymphangioma. Histopathology remains the gold standard for confirming the diagnosis.

Orbital RMS, being non-encapsulated, tends to invade surrounding orbital structures and adjacent osseous tissues, with potential intracranial extension, particularly in adults, even after chemoradiotherapy. The treatment typically involves surgical exenteration, either alone or in combination with chemoradiotherapy, depending on the stage of the disease. Recurrence and metastasis most often occur within the first three years post-treatment, with the lungs being the most common site for distant metastases [7]. Among the RMS subtypes, ARMS carries the worst prognosis. ARMS is thought to have a higher propensity for metastasizing to uncommon sites, including the breast, ovaries, testes, kidneys, and pancreas [6].

Metastasis to the pancreas is rare, and isolated pancreatic metastasis from RMS is seldom reported in the radiology literature. Staging of soft tissue sarcomas typically involves CECT of the chest and abdomen, along with whole-body bone scans. Although PET-CT is not always included in the initial workup, it can help detect metastases that may be missed on conventional imaging, such as pancreatic metastasis. PET-CT can reveal metastases with increased metabolic activity, or show lesions with marginal enhancement and central non-enhancement due to an under-perfused, hypercellular core, which may be mistaken for a cystic component [6]. Thus, adding PET-CT to standard imaging increases staging accuracy and enhances the detection of pancreatic metastasis in RMS patients. Adjustments in abdominal CT protocols may also improve pancreatic assessments, further aiding in detecting such metastases.

Nearly all reported cases of pancreatic metastasis from RMS involve the alveolar subtype. This may be linked to the PAX-FKHR fusion protein found in alveolar RMS, which stimulates the transcription of insulin-like growth factor I (IGF-I) and other growth factors, making the pancreas more susceptible to metastasis [6]. Consequently, it is crucial to consider pancreatic metastasis in patients with RMS, particularly those with the alveolar subtype. This case highlights the importance of recognizing isolated pancreatic metastasis in RMS patients, as its misidentification could lead to an erroneous downstaging of the disease, ultimately affecting treatment and outcomes.

## Conclusions

Orbital RMS is highly aggressive and should be considered in the differential diagnosis of rapidly progressive orbital masses. Although isolated pancreatic metastasis is a known complication of ARMS, it is seldom reported in the radiology literature. This case underscores a critical gap in recognizing and accurately staging the RMS patients with rare metastases, such as those involving the pancreas. The underrepresentation of isolated pancreatic metastases in the literature suggests a need for further research to refine imaging protocols, such as incorporating PET-CT more consistently and modifying abdominal CT protocols to enhance detection. Bridging this literature gap could improve staging accuracy and treatment outcomes in RMS patients.
